# The role of PPARγ in prostate cancer development and progression

**DOI:** 10.1038/s41416-022-02096-8

**Published:** 2022-12-12

**Authors:** Andrew Hartley, Imran Ahmad

**Affiliations:** 1 School of Cancer Sciences, College of Medical, Veterinary and Life Sciences, University of Glasgow, Glasgow, G61 1QH UK; 2 CRUK Beatson Institute, Garscube Estate, Switchback Road, Bearsden, Glasgow, G61 1BD UK

**Keywords:** Prostate cancer, Metabolomics

## Abstract

Advanced and metastatic prostate cancer is often incurable, but its dependency on certain molecular alterations may provide the basis for targeted therapies. A growing body of research has demonstrated that peroxisome proliferator-activated receptor gamma (PPARγ) is amplified as prostate cancer progresses. PPARγ has been shown to support prostate cancer growth through its roles in fatty acid synthesis, mitochondrial biogenesis, and co-operating with androgen receptor signalling. Interestingly, splice variants of PPARγ may have differing and contrasting roles. PPARγ itself is a highly druggable target, with agonists having been used for the past two decades in treating diabetes. However, side effects associated with these compounds have currently limited clinical use of these drugs in prostate cancer. Further understanding of PPARγ and novel techniques to target it, may provide therapies for advanced prostate cancer.

## Background

Prostate cancer (PC) is the most common malignancy in men and the second leading cause of cancer death in men in the developed world [1]. While the early stages of this disease are curable, the advanced and metastatic forms of this disease have no curative options and account for the vast majority of deaths from PC.

The aetiology of PC is ill-defined and, due to the highly heterogenous nature of this disease, identifying true drivers of PC remains challenging. For this reason, for the vast majority of patients, the only targeted treatment available is androgen-deprivation therapy (ADT), which inhibits androgen receptor (AR) activity [2]. However, response to ADT is time-limited, and with time, resistance occurs [3]. Patients who develop resistance to ADT are said to have castrate-resistant PC for which there is no curative therapy. Identifying targetable mutations in advanced PC will help these patients who have the greatest unmet need.

PC growth is intrinsically linked to fatty acid and cholesterol biosynthesis [4]. Many key regulators of these metabolic pathways are overexpressed in PC and are implicated as oncogenic drivers of the disease [5].

Peroxisome proliferator-activated receptor (PPAR) are members of the nuclear hormone receptor superfamily [6]. There are three subtypes of PPAR that have been identified, α, β (also referred to as δ), and γ. PPARα and PPARβ have not been well studied in cancer and their roles in PC progression are not well understood. This review will therefore focus on PPARγ and its known roles in PC as it is the most well defined.

PPARs function by binding DNA elements called PPAR response elements (PPREs) and promoting gene expression of the adjacent genes [7]. This PPRE sequence was previously identified to be two AGGTCA repeats with a single-nucleotide spacer [7]. However, more recent studies identified that PPAR subtypes likely have preferences for different sequences and variations of this previously identified PPRE [8]. These differences allowed identification of novel PPAR target genes and unveiled greater complexity in their regulation.

PPARγ’s most well-defined role is as a master regulator of adipogenesis, where it controls lipid metabolism and insulin sensitivity [9, 10]. PPARγ is also essential for adipocyte differentiation and maintenance [11–13]. Due to PPARγ’s role in lipogenesis, many ligands are fatty acids, including polyunsaturated fatty acids, branched chain fatty acids, and saturated fatty acids [6, 14, 15]. Synthetic ligands have also been generated to activate PPARγ as agonists. This class of drugs, the thiazolidinediones (TZDs), activate PPARγ and induce fatty acid uptake from the blood into peripheral fat thereby improving insulin sensitivity [16]. As such, these drugs are used clinically in treating type 2 diabetes mellitus (T2DM) [17].

**Fig. 1:**
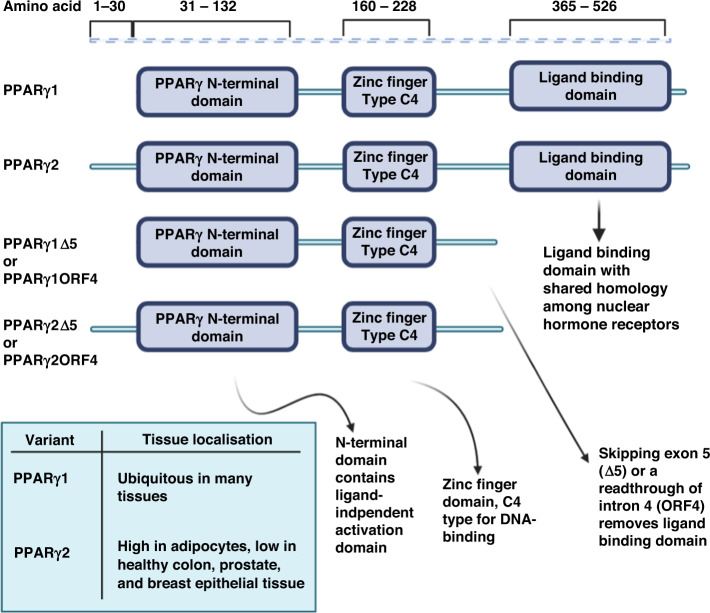
Comparison of PPARγ splice variants. Various PPARγ splice variants can be produced by alternative splicing events including exon skipping and readthrough. These variants each have unique structures and tissues specificity. The most well studied variants PPARγ1 and PPARγ1 however are of the most interest in prostate cancer currently.

PPARγ has two well-studied splice variants in PC, PPARγ1 and PPARγ2 (Fig. 1). The key structural difference in these variants is in the N-terminus, where PPARγ2 has an additional 30 amino acids [18]. Contained in the N-terminus is a ligand-independent activation domain, and PPARγ2 was demonstrated to have a fivefold greater ligand-independent activity likely as an impact of these additional 30 amino acids [19]. Each of these variants also show differences in tissue specificity, with PPARγ1 being highly ubiquitous in many tissues, whereas PPARγ2 was originally only believed to be found in adipocytes [6, 20]. Later studies identified PPARγ2 to be expressed at low levels in healthy breast, colon, bladder, and prostate tissue [21, 22]. Other PPARγ splice variants have been identified with a truncated sequence resulting in a non-functional ligand-binding domain, though these variants still retain DNA-binding capacity [23]. These truncated variants are produced either by skipping exon 5 in the case of y1Δ5 and y1Δ5 variants or by readthrough of exon 4 in the case of y1ORF4 and y2ORF4 [23–25]. These variants are suggested to negatively regulate PPARγ by competing for binding sites while being unable to activate gene expression themselves [23]. However, these variants have not been studied in PC.

## Intracellular signalling of PPARγ in prostate cancer

Besides regulating systemic responses such as insulin sensitivity, PPARγ also regulates intracellular metabolism and signalling events which have been implicated in cancer. Our group has previously identified that an elevation of *Ppar*γ*1* expression in a phosphatase and tensin homology (*Pten*) null PC murine model led to an acceleration in prostate tumourigenesis and increased tumour weight at clinical endpoint [26]. These mice with elevated *Ppar*γ*1* also had a reduced survival and increased incidence of metastasis, both locally to pelvic lymph nodes and distally to the lungs. This elevation in *Ppar*γ*1* correlated with an increase in lipid synthesis machinery, including fatty acid synthase (FASN) and ATP citrate lyase (ACYL). We later identified the mechanism by which PPARγ can drive aggressive disease through an AKT serine/threonine kinase 3 (AKT3), PPARG coactivator 1 alpha (PGC1α), chromosome maintenance region 1 (CRM1) axis [27]. This culminates in PPARγ controlling mitochondrial biogenesis through this axis, with an elevation in PPARγ leading to an increase in mitochondrial mass capable of driving advanced disease [27]. PPARγ’s regulation of mitochondrial biogenesis has been previously observed in adipocyte, neuronal, and bladder epithelial cell lines, though this is the first time this process has been linked to PC [28–30]. Interestingly, overexpression of PPARγ in PC3-M did not alter growth in 2D. However, spheroids of PC3-M were cultured in 3D with matrigel and overexpression of PPARγ increased an epithelial–mesenchymal transition (EMT) phenotype. EMT markers were also increased following PPARγ overexpression. These findings highlight how PPARγ transcriptional targets can be hijacked by PC and used to drive aggressive disease.

However, a previous study investigating PPARγ variants suggested that these effects are unique to certain variants. This study utilised an in vivo knockout of PPARγ and then restoration of either PPARγ1 or PPARγ2 to study the specific effects of each variant [31]. This showed that PPARγ1 and PPARγ2 both reduced lipogenesis in vivo by reducing expression of key lipogenic regulators including FASN and acetyl-CoA carboxylase alpha (ACACA) [31]. Furthermore, PPARγ1 was demonstrated to downregulate stearoyl-CoA desaturase 1 (SCD1), while PPARγ2 upregulated SCD1. As SCD1 is a key regulator of fatty acid metabolism, this suggests a variant-specific function [31]. These findings suggest that both PPARγ variants reduce lipogenesis, whereas our own data showed increased PPARγ1, increased FASN and ACYL. This discrepancy may be due to *Pten* alterations with our genetically engineered mouse model employing *Pten* loss as a driving mutation [26].

PPARγ variants also had differential impacts on cellular signalling, influencing prostate epithelial differentiation, with an increase in PPARγ2 producing a basal-like phenotype, but not PPARγ1, which remained luminal [31]. PPARγ1 was also shown to produce an adenocarcinoma subtype, whereas PPARγ2 developed acini resembling normal prostate glands. This is validated by our own work which had demonstrated that increased *Ppar*γ*1* in vivo elevated prostate tumourigenesis. The differences between these variants are summarised in Table 1.

**Table 1:** Comparison of PPARγ variants in regulating prostate cancer signalling and development.

Prostate epithelial characteristics	PPARγ1	PPARγ2
Lipogenisis (FASN, ACACA)	↓	↓
Fatty acid metabolism (SCD1)	↓	↑
Differentiation	Luminal	Basal
Histology	Adenocarcinoma	Benign
AR signalling	↓	↑

*PPARγ1* and *PPARγ2* peroxisome proliferator-activated receptor gamma variants 1 and 2, *FASN* fatty acid synthase, *ACACA* acetyl-CoA carboxylase alpha, *SCD1* stearoyl-CoA desaturase 1.

PPARγ2 was later investigated and shown once again to be an inhibitor of PC growth [22]. PPARγ2 expression was shown to be decreased in PC cell lines LNCaP, PC3, and DU145 compared to a normal prostate cell line NHPrE1. Furthermore, overexpression of PPARγ2 decreased colony forming, migration, invasion, and proliferation in PC3 and LNCaP. Mechanistically, this was shown to be caused by PPARγ2 upregulating expression of A-kinase anchor protein 12 (AKAP12), which in turn downregulated AKT signalling.

These findings suggest a more complex context-dependent function for PPARγ variants. These effects may be caused by PPARγ2’s enhanced ligand-independent activity and its own unique functionality compared to PPARγ1. Other studies have also identified differing ligand-dependent activity with PPARγ2 having enhanced transcriptional activity compared to PPARγ1 at low ligand concentrations [32]. This difference was attributed to PPARγ2 interacting more strongly with the DRIP/TRAP/ARC complex, which coactivates nuclear receptor signalling [32].

The distinctions could be further compounded in a disease setting due to the dynamic expression of PPARγ variants themselves [33]. During adipogenesis, PPARγ1 is ubiquitously expressed throughout adipocyte differentiation, whereas PPARγ2 expression dynamically changes [33]. Discrepancies in the activity of these variants, their expression, and their interacting partners could contribute to the differences observed between these variants in PC.

## PPARγ and androgen receptor (AR)

**Fig. 2:**
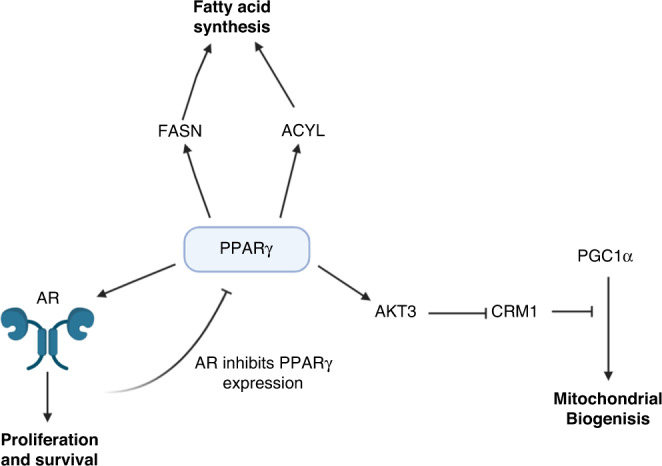
PPARγ signalling that positively regulate prostate cancer growth. PPARγ peroxisome proliferator-activated receptor gamma, FASN fatty acid synthase, ACYL ATP citrate lyase, AR androgen receptor, AKT3 AKT serine/threonine kinase 3, CRM1 chromosome region maintenance 1, PCG1α PPARG coactivator 1 alpha.

PPARγ has also been shown to interact with key oncogenic signalling proteins in PC such as AR [34] (Fig. 2). This was first discovered due to the observation that long term use of warfarin, an anticoagulant commonly used clinically, reduces the risk of PC [35]. By treating mice with warfarin and performing RNA-Sequencing, PPARγ was identified to be inhibited following warfarin treatment, which in turn inhibited AR signalling [34]. This interaction of PPARγ and AR may also demonstrate another distinction between PPARγ1 and PPARγ2 isoforms. An in vivo study showed that PPARγ1 reduced AR transcriptional activity, whereas PPARγ2 increased AR transcriptional activity [31]. This may suggest that warfarin acts on PPARγ2 to inhibit its pro-AR signalling, and not on PPARγ1. Interestingly, AR was also demonstrated to negatively regulate PPARγ expression, indicating a potential negative feedback loop between these two proteins and isoforms [36]. These findings may suggest that in a castrate-setting following ADT, PPARγ expression is elevated to support cell growth when AR is inactive. Interestingly, castrate-sensitive LNCaP cells demonstrate an inhibition of AR activity following PPARγ agonism by ciglitazone and rosiglitazone, whereas castrate-resistant C4-2 see an activation of AR activity with the same compounds [37]. This was further demonstrated to be a PPARγ-dependent increase in AR activity. Despite this, both C4-2 and LNCaP have impaired growth when treated with ciglitazone and rosiglitazone, though this effect may not be entirely PPARγ-dependent [38]. These data may suggest a unique role for PPARγ and its variants in a castrate-resistant PC.

## PPARγ agonism by TZDs and its role in prostate cancer

TZDs are a class of drugs that bind and activate PPARγ and are commonly used clinically in the treatment of T2DM [39, 40]. Clinical data from long-term usage of TZD drugs have informed us about the role of PPARγ activation in the development of PC. One study suggested that TZDs have no impact on PC incidence and even reduced the incidence in lung cancer [41]. This was later confirmed in a meta-analysis which found a slight trend toward TZDs reducing PC incidence [42]. It has also been shown that diabetic patients with PC have an improved survival when treated with a combination of TZDs and metformin [43]. These effects may be due to a direct impact of these drugs on PC tissue, suggesting that PPARγ activation may reduce the risk of PC development. Alternatively, TZDs and metformin could effect PC indirectly by targeting liver and adipose tissue, thereby improving the overall metabolic health of the patient.

One potential explanation for a direct effect of TZDs on PC would be that TZDs impair PC growth. This was demonstrated with one TZD, troglitatone, which could impede growth of PC cell line PC3, and reduce prostate biomarker, prostate-specific antigen (PSA), expression in LNCaP [44, 45]. However, the impact of TZDs on PC growth has been suggested to be a PPARγ-independent effect [38]. This is apparent since the dose used in vitro is far higher than that required to activate PPARγ, and appears to impair prostate cancer cell growth [46]. Thus, the reduction of PC growth by TZDs has been attributed to a reduction in c-Myc expression, and extracellular signal-regulated kinase (ERK) phosphorylation though PPARγ-independent mechanisms [47, 48]. It has even been suggested that a sub-lethal dose of TZDs, which only activates PPARγ, can promotes cell survival. In all, this suggests that TZDs are unlikely to have a significant impact on PC development, and any reduction in PC growth may be in a PPARγ-dependent manner.

## PPARγ alterations in prostate cancer

By using a tissue microarray (TMA) and staining for PPARγ immunohistochemically, Rogenhofer et al. found that protein levels of PPARγ were increased in advanced PC compared to both low-risk PC and benign prostate hyperplasia (BPH) [49]. A separate study stained clinical samples for PPARγ by immunohistochemistry (IHC) and found protein levels to be increased in PC and prostate intraepithelial neoplasia (PIN), compared to BPH and normal prostatic tissue [50]. Finally, our own group found that PPARγ levels correlated with Gleason grades, increasing in grades 3-5 compared to BPH [26]. We determined the correlation of PPARγ levels and patient survival using a TMA. Interestingly, PPARγ levels alone were not a prognostic indicator; however, high PPARγ levels in patients with a low PTEN level (2 years vs 7 years median survival) or high phospho-AKT cohorts (2.1 years vs 6.3 years median survival) led to a reduction in overall survival. This suggests an interplay between PPARγ and phosphoinositide 3-kinase–AKT signalling [26].

**Fig. 3:**
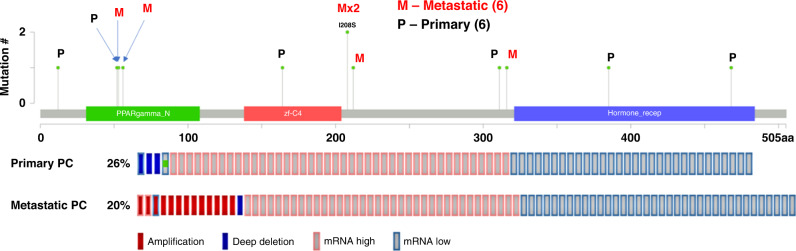
PPARγ alterations in PC (CBioPortal). PPARγ alterations visualised using CBioPortal [42, 43]. Prostate adenocarcinoma TCGA (51) was used as the primary prostate cancer cohort [40]. Metastatic prostate adenocarcinoma (SU2C/PCF) was used as the metastatic prostate cancer cohort [41]. For mRNA dysregulation, a *z*-score of >1.2 was used as a threshold relative to all samples.

Using CBioPortal, we can visualise the alterations of PPARγ in PC (Fig. 3) [51–54]. PPARγ mutations are most often missense mutations, with equal instances of mutations being observed in primary or metastatic clinical samples. Interestingly there is no consistency with mutations in certain domains despite missense mutations being likely to lead to amino acid substitutions. However, when we look at copy number variation, this demonstrates that metastatic PC has a higher instance of amplification of PPARγ compared to primary PC. mRNA dysregulation occurs at similar rates in both primary and metastatic PC with some cases upregulating and downregulating PPARγ expression.

As PPARγ2 has an additional 30 amino acids in its N-terminal compared to PPARγ1, it has been observed to have its own specific polymorphisms in that region with codon 12 having a missense mutation causing a substitution of proline to alanine. This polymorphism has an estimated frequency of 0.12 in a random Caucasian population [55]. This Pro12Ala substitution is associated with an increased incidence of colorectal cancer and breast cancer [56, 57]. However, in investigating this Pro12Ala in PC, this polymorphism is not associated with increased PC risk or more aggressive disease [58]. This study, however, was performed on a Finnish population and would need to be replicated with more diverse population to be conclusive.

These findings appear consistent in showing that total PPARγ levels increases from BPH to PIN to PC and increase with Gleason grade. Further investigation into the changes PPARγ variants between these stages will improve our understanding of the interplay of these variants in PC.

## PPARγ and diet in prostate cancer

Obese men (body mass index >30) with PC have a higher PC-specific morality as well as a higher all-cause mortality when compared to ‘normal weight’ men (body mass index <25) [59, 60]. While understanding the interplay of obesity and PC is complex, some studies have attempted to identify the role of dietary fatty acids and PC. As PPARγ is a receptor for fatty acids in prostate epithelial cells, it has been implicated in the association of fatty acids and PC.

One study utilised an in vivo murine model, where mice were fed a diet rich in saturated fatty acids, compared to a poly-unsaturated fatty acid diet [61]. Saturated fatty acid rich diet led to mice having an enlarged prostate, with an increase in prostate epithelial volume and decreased lumen size of prostate glands due of epithelial hyperplasia. RNA-Seq showed that saturated fatty acid rich diet modulated the immune system and systemic inflammation. This diet let to an increase in pro-inflammatory cytokines and prostatitis, and to an inflammation of the prostate itself. Interestingly, in comparison, polyunsaturated fatty acids had opposing effects to saturated fatty acids. Poly-unsaturated fatty acids have been suggested previously to have a protective role against cancer development [62]. These effects were shown in vitro to be PPARγ-dependent, with poly-unsaturated fatty acids decreasing proliferation and elevating apoptosis of breast cancer cells [63, 64]. In PC, patients are often observed to have elevated free fatty acids in their serum [65]. Furthermore, elevated fatty acids increased PPARγ levels and increased proliferation and invasion of PC3 and DU145 in a PPARγ-dependent manner [65].

These studies may therefore implicate PPARγ in being effector, by which a saturated fatty acid rich diet can elevate mortality. However, as both saturated and poly-unsaturated fatty acids have been identified as ligands of PPARγ this could also suggest a differential response in a ligand-dependent manner [6, 14, 15]. Further understanding PPARγ and dietary fatty acids may allow for a targeted therapy in obese patients with PC.

## Future directions

PPARγ was first identified in 1994 and TZDs rosiglitazone and pioglitazone were marketed in 1999 for treatment of T2DM [66–68]. Since then, two decades of scientific research have improved our understanding of PPARγ, and ongoing research continues to highlight the role of PPARγ in prostate cancer. While previously thought to be adipocyte specific, PPARγ2 has emerged as a unique ‘tumour suppressor’ in comparison to a more ‘oncogenic’ PPARγ1. However, the context-dependent complexities regarding the interactions of these two variants and their roles in the development and advancement of PC remain unclear.

Clinical data confirms that PPARγ levels rise as PC develops, and PC can develop a dependency on PPARγ for lipogenesis and mitochondrial biogenesis, particularly in vivo. This may suggest that antagonism against PPARγ may be a viable therapeutic option for inhibiting PC development. PPARγ antagonists such as betulinic acid have been developed and used in murine models as potential therapies for diabetes which avoid the side-effects associated with PPARγ antagonism [69]. Small molecular inhibitors however may have even fewer side effects. Use of one small molecule, T0070907, was shown to impair growth of PC cell lines LCP and PC3 in vitro [70]. LCP cells were also used in a xenograft and treated with T0070907 whereupon 4/7 tumours could no longer be detected indicating a complete regression. This inhibition of growth was shown to be through conventional PPARγ signalling with fatty acid synthesis genes FASN and ACACA being downregulated, as well as AR-dependent pathways, suggesting that the PPARγ–AR interactions can be targeted [70]. This same small molecular inhibitor was shown to inhibit growth of breast cancer cell lines through PPARγ-dependent pathways and was also suggested to impair MAPK signalling [71].

We have also demonstrated the use of PPARγ antagonist GW9662 as an inhibitor of PC growth [26]. Use of GW9662 impaired metastasis of a PC3 orthograft, as well impairing growth and colony forming in in vitro. GW9662 was later also shown to impair growth of a PC3-M xenograft [72]. However, GW9662 may not be a suitable drug for clinical use, due to its systemic effects on PPARγ leading to a reduction of whole-body visceral fat indicating adipogenesis may be affected [73].

These data suggest that antagonism of PPARγ may provide a therapeutic target for PC, particularly the advanced stages which seem to become increasingly reliant on its activity. Due to the apparent different roles of the PPARγ variants in PC, therapeutics which specifically target different isoforms may also have a profound effect on PC. This would allow the inhibition of oncogenic PPARγ1 signalling while retaining any tumour suppressing activity of PPARGγ2. Further research into PPARγ antagonism, and the interactions of PPARγ variants will elucidate the next steps in exploiting PC dependency on PPARγ.

## References

[1] Siegel RL, Miller KD, Jemal A (2015). Cancer statistics, 2015. CA Cancer J Clin.

[2] Huggins C, Hodges CV (1972). Studies on prostatic cancer. I. The effect of castration, of estrogen and androgen injection on serum phosphatases in metastatic carcinoma of the prostate. CA Cancer J Clin.

[3] Maitland NJ. Resistance to antiandrogens in prostate cancer: is it inevitable, intrinsic or induced? Cancers. 2021;13:327. 10.3390/cancers13020327 PMC7829888 33477370

[4] Wang X, Sun B, Wei L, Jian X, Shan K, He Q (2022). Cholesterol and saturated fatty acids synergistically promote the malignant progression of prostate cancer. Neoplasia..

[5] Wu X, Daniels G, Lee P, Monaco ME (2014). Lipid metabolism in prostate cancer. Am J Clin Exp Urol.

[6] Rosen ED, Spiegelman BM (2001). PPARgamma: a nuclear regulator of metabolism, differentiation, and cell growth. J Biol Chem.

[7] Ijpenberg A, Jeannin E, Wahli W, Desvergne B (1997). Polarity and specific sequence requirements of peroxisome proliferator-activated receptor (PPAR)/retinoid X receptor heterodimer binding to DNA: a functional analysis of the malic enzyme gene PPAR response element. J Biol Chem.

[8] Tzeng J, Byun J, Park JY, Yamamoto T, Schesing K, Tian B (2015). An ideal PPAR response element bound to and activated by PPARα. PLoS ONE.

[9] Ahmadian M, Suh JM, Hah N, Liddle C, Atkins AR, Downes M (2013). PPARγ signaling and metabolism: the good, the bad and the future. Nat Med.

[10] Rosen ED, Walkey CJ, Puigserver P, Spiegelman BM (2000). Transcriptional regulation of adipogenesis. Genes Dev.

[11] Rosen ED, Sarraf P, Troy AE, Bradwin G, Moore K, Milstone DS (1999). PPAR gamma is required for the differentiation of adipose tissue in vivo and in vitro. Mol Cell.

[12] Wu Z, Rosen ED, Brun R, Hauser S, Adelmant G, Troy AE (1999). Cross-regulation of C/EBP alpha and PPAR gamma controls the transcriptional pathway of adipogenesis and insulin sensitivity. Mol Cell.

[13] Rosen ED, Hsu CH, Wang X, Sakai S, Freeman MW, Gonzalez FJ (2002). C/EBPalpha induces adipogenesis through PPARgamma: a unified pathway. Genes Dev.

[14] Trombetta A, Maggiora M, Martinasso G, Cotogni P, Canuto RA, Muzio G (2007). Arachidonic and docosahexaenoic acids reduce the growth of A549 human lung-tumor cells increasing lipid peroxidation and PPARs. Chem Biol Interact.

[15] Heim M, Johnson J, Boess F, Bendik I, Weber P, Hunziker W (2002). Phytanic acid, a natural peroxisome proliferator-activated receptor (PPAR) agonist, regulates glucose metabolism in rat primary hepatocytes. FASEB J.

[16] Hauner H (2002). The mode of action of thiazolidinediones. Diabetes Metab Res Rev.

[17] Kletzien RF, Clarke SD, Ulrich RG (1992). Enhancement of adipocyte differentiation by an insulin-sensitizing agent. Mol Pharmacol.

[18] Tontonoz P, Hu E, Graves RA, Budavari AI, Spiegelman BM (1994). mPPAR gamma 2: tissue-specific regulator of an adipocyte enhancer. Genes Dev.

[19] Werman A, Hollenberg A, Solanes G, Bjorbaek C, Vidal-Puig AJ, Flier JS (1997). Ligand-independent activation domain in the N terminus of peroxisome proliferator-activated receptor gamma (PPARgamma). Differential activity of PPARgamma1 and -2 isoforms and influence of insulin. J Biol Chem.

[20] Fajas L, Auboeuf D, Raspe E, Schoonjans K, Lefebvre AM, Saladin R (1997). The organization, promoter analysis, and expression of the human PPARgamma gene. J Biol Chem.

[21] Tran L, Bobe G, Arani G, Zhang Y, Zhang Z, Shannon J, et al. Diet and PPARG2 Pro12Ala polymorphism interactions in relation to cancer risk: a systematic review. Nutrients. 2021;13:261. 10.3390/nu13010261 PMC7831057 33477496

[22] Li F, Lu T, Liu D, Zhang C, Zhang Y, Dong F (2021). Upregulated PPARG2 facilitates interaction with demethylated AKAP12 gene promoter and suppresses proliferation in prostate cancer. Cell Death Dis.

[23] Aprile M, Cataldi S, Ambrosio MR, D’Esposito V, Lim K, Dietrich A (2018). PPARγΔ5, a naturally occurring dominant-negative splice isoform, impairs PPARγ function and adipocyte differentiation. Cell Rep.

[24] Sabatino L, Casamassimi A, Peluso G, Barone MV, Capaccio D, Migliore C (2005). A novel peroxisome proliferator-activated receptor gamma isoform with dominant negative activity generated by alternative splicing. J Biol Chem.

[25] Mukha A, Kalkhoven E, van Mil SWC (2021). Splice variants of metabolic nuclear receptors: Relevance for metabolic disease and therapeutic targeting. Biochimica et Biophysica Acta Mol Basis Dis.

[26] Ahmad I, Mui E, Galbraith L, Patel R, Tan EH, Salji M (2016). Sleeping Beauty screen reveals Pparg activation in metastatic prostate cancer. Proc Natl Acad Sci USA.

[27] Galbraith LCA, Mui E, Nixon C, Hedley A, Strachan D, MacKay G (2021). PPAR-gamma induced AKT3 expression increases levels of mitochondrial biogenesis driving prostate cancer. Oncogene..

[28] Miglio G, Rosa AC, Rattazzi L, Collino M, Lombardi G, Fantozzi R (2009). PPARgamma stimulation promotes mitochondrial biogenesis and prevents glucose deprivation-induced neuronal cell loss. Neurochem Int.

[29] Rong JX, Klein JL, Qiu Y, Xie M, Johnson JH, Waters KM (2011). Rosiglitazone induces mitochondrial biogenesis in differentiated murine 3T3-L1 and C3H/10T1/2 adipocytes. PPAR Res..

[30] Liu C, Tate T, Batourina E, Truschel ST, Potter S, Adam M (2019). Pparg promotes differentiation and regulates mitochondrial gene expression in bladder epithelial cells. Nat Commun.

[31] Strand DW, Jiang M, Murphy TA, Yi Y, Konvinse KC, Franco OE (2012). PPARγ isoforms differentially regulate metabolic networks to mediate mouse prostatic epithelial differentiation. Cell Death Dis.

[32] Mueller E, Drori S, Aiyer A, Yie J, Sarraf P, Chen H (2002). Genetic analysis of adipogenesis through peroxisome proliferator-activated receptor gamma isoforms. J Biol Chem.

[33] Aprile M, Ambrosio MR, D’Esposito V, Beguinot F, Formisano P, Costa V (2014). PPARG in human adipogenesis: differential contribution of canonical transcripts and dominant negative isoforms. PPAR Res.

[34] Tew BY, Hong TB, Otto-Duessel M, Elix C, Castro E, He M (2017). Vitamin K epoxide reductase regulation of androgen receptor activity. Oncotarget..

[35] Patel S, Singh R, Preuss CV, Patel N. Warfarin. Treasure Island (FL): StatPearls Publishing LLC.; 2022.

[36] Olokpa E, Bolden A, Stewart LV (2016). The androgen receptor regulates PPARγ expression and activity in human prostate cancer cells. J Cell Physiol.

[37] Moss PE, Lyles BE, Stewart LV (2010). The PPARγ ligand ciglitazone regulates androgen receptor activation differently in androgen-dependent versus androgen-independent human prostate cancer cells. Exp Cell Res.

[38] Galbraith L, Leung HY, Ahmad I (2018). Lipid pathway deregulation in advanced prostate cancer. Pharmacol Res.

[39] Durbin RJ (2004). Thiazolidinedione therapy in the prevention/delay of type 2 diabetes in patients with impaired glucose tolerance and insulin resistance. Diabetes Obes Metab.

[40] Spiegelman BM (1998). PPAR-gamma: adipogenic regulator and thiazolidinedione receptor. Diabetes..

[41] Govindarajan R, Ratnasinghe L, Simmons DL, Siegel ER, Midathada MV, Kim L (2007). Thiazolidinediones and the risk of lung, prostate, and colon cancer in patients with diabetes. J Clin Oncol.

[42] Nath M, Nath S, Choudhury Y (2021). The impact of thiazolidinediones on the risk for prostate cancer in patients with type 2 diabetes mellitus: a review and meta-analysis. Meta Gene.

[43] He XX, Tu SM, Lee MH, Yeung SJ (2011). Thiazolidinediones and metformin associated with improved survival of diabetic prostate cancer patients. Ann Oncol.

[44] Kubota T, Koshizuka K, Williamson EA, Asou H, Said JW, Holden S (1998). Ligand for peroxisome proliferator-activated receptor gamma (troglitazone) has potent antitumor effect against human prostate cancer both in vitro and in vivo. Cancer Res.

[45] Hisatake JI, Ikezoe T, Carey M, Holden S, Tomoyasu S, Koeffler HP (2000). Down-regulation of prostate-specific antigen expression by ligands for peroxisome proliferator-activated receptor gamma in human prostate cancer. Cancer Res.

[46] Wang YL, Frauwirth KA, Rangwala SM, Lazar MA, Thompson CB (2002). Thiazolidinedione activation of peroxisome proliferator-activated receptor gamma can enhance mitochondrial potential and promote cell survival. J Biol Chem.

[47] Akinyeke TO, Stewart LV (2011). Troglitazone suppresses c-Myc levels in human prostate cancer cells via a PPARγ-independent mechanism. Cancer Biol Ther.

[48] Bolden A, Bernard L, Jones D, Akinyeke T, Stewart LV (2012). The PPAR gamma agonist troglitazone regulates Erk 1/2 phosphorylation via a PPARγ-independent, MEK-dependent pathway in human prostate cancer cells. PPAR Res.

[49] Rogenhofer S, Ellinger J, Kahl P, Stoehr C, Hartmann A, Engehausen D (2012). Enhanced expression of peroxisome proliferate-activated receptor gamma (PPAR-γ) in advanced prostate cancer. Anticancer Res.

[50] Segawa Y, Yoshimura R, Hase T, Nakatani T, Wada S, Kawahito Y (2002). Expression of peroxisome proliferator-activated receptor (PPAR) in human prostate cancer. Prostate..

[51] The Cancer Genome Atlas Research Network. The molecular taxonomy of primary prostate cancer. Cell. 2015;163:1011–25. 10.1016/j.cell.2015.10.025 PMC4695400 26544944

[52] Abida W, Cyrta J, Heller G, Prandi D, Armenia J, Coleman I (2019). Genomic correlates of clinical outcome in advanced prostate cancer. Proc Natl Acad Sci USA.

[53] Cerami E, Gao J, Dogrusoz U, Gross BE, Sumer SO, Aksoy BA (2012). The cBio cancer genomics portal: an open platform for exploring multidimensional cancer genomics data. Cancer Discov.

[54] Gao J, Aksoy BA, Dogrusoz U, Dresdner G, Gross B, Sumer SO (2013). Integrative analysis of complex cancer genomics and clinical profiles using the cBioPortal. Sci Signal.

[55] Hasstedt SJ, Ren QF, Teng K, Elbein SC (2001). Effect of the peroxisome proliferator-activated receptor-gamma 2 pro(12)ala variant on obesity, glucose homeostasis, and blood pressure in members of familial type 2 diabetic kindreds. J Clin Endocrinol Metab.

[56] Wang W, Shao Y, Tang S, Cheng X, Lian H, Qin C (2015). Peroxisome proliferator-activated receptor-γ (PPARγ) Pro12Ala polymorphism and colorectal cancer (CRC) risk. Int J Clin Exp Med.

[57] Mao Q, Guo H, Gao L, Wang H, Ma X (2013). Peroxisome proliferator-activated receptor γ2 Pro12Ala (rs1801282) polymorphism and breast cancer susceptibility: a meta-analysis. Mol Med Rep.

[58] Paltoo D, Woodson K, Taylor P, Albanes D, Virtamo J, Tangrea J (2003). Pro12Ala polymorphism in the peroxisome proliferator-activated receptor-gamma (PPAR-γ) gene and risk of prostate cancer among men in a large cancer prevention study. Cancer Lett.

[59] Freedland SJ, Aronson WJ (2004). Examining the relationship between obesity and prostate cancer. Rev Urol.

[60] Rivera-Izquierdo M, Pérez de Rojas J, Martínez-Ruiz V, Pérez-Gómez B, Sánchez MJ, Khan KS, et al. Obesity as a risk factor for prostate cancer mortality: a systematic review and dose-response meta-analysis of 280,199 patients. Cancers. 2021;13:4169. 10.3390/cancers13164169 PMC8392042 34439328

[61] Ferrucci D, Silva SP, Rocha A, Nascimento L, Vieira AS, Taboga SR (2019). Dietary fatty acid quality affects systemic parameters and promotes prostatitis and pre-neoplastic lesions. Sci Rep.

[62] Edwards IJ, O’Flaherty JT (2008). Omega-3 fatty acids and PPARgamma in cancer. PPAR Res.

[63] Sun H, Berquin IM, Owens RT, O’Flaherty JT, Edwards IJ (2008). Peroxisome proliferator-activated receptor gamma-mediated up-regulation of syndecan-1 by n-3 fatty acids promotes apoptosis of human breast cancer cells. Cancer Res.

[64] Edwards IJ, Berquin IM, Sun H, O’Flaherty JT, Daniel LW, Thomas MJ (2004). Differential effects of delivery of omega-3 fatty acids to human cancer cells by low-density lipoproteins versus albumin. Clin Cancer Res.

[65] Ha X, Wang J, Chen K, Deng Y, Zhang X, Feng J (2020). Free fatty acids promote the development of prostate cancer by upregulating peroxisome proliferator-activated receptor gamma. Cancer Manag Res.

[66] Kliewer SA, Forman BM, Blumberg B, Ong ES, Borgmeyer U, Mangelsdorf DJ (1994). Differential expression and activation of a family of murine peroxisome proliferator-activated receptors. Proc Natl Acad Sci USA.

[67] Rosen CJ (2007). The rosiglitazone story—lessons from an FDA Advisory Committee meeting. N Engl J Med.

[68] Sohda T, Kawamatsu Y, Fujita T, Meguro K, Ikeda H (2002). [Discovery and development of a new insulin sensitizing agent, pioglitazone]. Yakugaku Zasshi.

[69] Brusotti G, Montanari R, Capelli D, Cattaneo G, Laghezza A, Tortorella P (2017). Betulinic acid is a PPARγ antagonist that improves glucose uptake, promotes osteogenesis and inhibits adipogenesis. Sci Rep.

[70] Elix CC, Salgia MM, Otto-Duessel M, Copeland BT, Yoo C, Lee M (2020). Peroxisome proliferator-activated receptor gamma controls prostate cancer cell growth through AR-dependent and independent mechanisms. Prostate..

[71] Zaytseva YY, Wallis NK, Southard RC, Kilgore MW (2011). The PPARgamma antagonist T0070907 suppresses breast cancer cell proliferation and motility via both PPARgamma-dependent and -independent mechanisms. Anticancer Res.

[72] Al-Jameel W, Gou X, Forootan SS, Al Fayi MS, Rudland PS, Forootan FS (2017). Inhibitor SBFI26 suppresses the malignant progression of castration-resistant PC3-M cells by competitively binding to oncogenic FABP5. Oncotarget..

[73] Nakano R, Kurosaki E, Yoshida S, Yokono M, Shimaya A, Maruyama T (2006). Antagonism of peroxisome proliferator-activated receptor gamma prevents high-fat diet-induced obesity in vivo. Biochem Pharmacol.

